# Intra- and Interobserver Variability of Shear Wave Elastography in Rectal Cancer

**DOI:** 10.3390/cancers14112633

**Published:** 2022-05-26

**Authors:** Martina Kastrup Loft, Malene Roland Vils Pedersen, Peter Grimm, Andreas Hoffmann Lauritzen, Claus Dam, Søren Rafael Rafaelsen

**Affiliations:** 1 Department of Radiology, Lillebaelt Hospital, Clinical Cancer Centre, University Hospital of Southern Denmark, Beriderbakken 4, 7100 Vejle, Denmark; malene.roland.vils.pedersen@rsyd.dk (M.R.V.P.); peter.grimm2@rsyd.dk (P.G.); andreas.hoffmann.lauritzen@rsyd.dk (A.H.L.); claus.dam@rsyd.dk (C.D.); soeren.rafael.rafaelsen@rsyd.dk (S.R.R.); 2 Department of Regional Health Research, University of Southern Denmark, Campusvej 55, 5000 Odense, Denmark; 3 Danish Colorectal Cancer Center South, Lillebaelt Hospital, University Hospital of Southern Denmark, 7100 Vejle, Denmark

**Keywords:** shear wave elastography, ultrasound, rectal neoplasms, interobserver, reproducibility

## Abstract

**Simple Summary:**

For a diagnostic tool to be important, there must be a high level of agreement between different observers. If not, the reliability of the measurement can not be considered valid. Previous studies have evaluated the reproducibility of tissue stiffness measurements of rectal tumors, but only on previously obtained images. Therefore, we aimed to investigate the issue in a clinical setting and found a high level of agreement between observers.

**Abstract:**

Background: Endorectal ultrasound (ERUS) is an important tool when evaluating complex rectal adenomas and rectal cancer, and the accuracy is improved by adding elastography measurements. A high interobserver agreement is imperative in clinical practice. Therefore, the aim of this study was to evaluate interobserver agreement assessed on real-time images. Additionally, we investigated the intra- and interobserver agreement between experienced and inexperienced observers. Materials and methods: We prospectively included patients referred to an ERUS at the Department of Radiology with a complex rectal polyp or suspected rectal malignancy. Two operators independently scanned each patient in turn. Furthermore, four observers assessed previously obtained images using three different methods for placing the region of interest (ROI). Three months later, the four observers reassessed the images to assess intraobserver variability. Results: A total of 19 patients were included for live assessment. Agreement of tumor classification was substantial for T stage (kappa: 0.86) and fair for N stage (kappa: 0.73), with an absolute agreement for T and N stages of 84% and 89%, respectively. Agreement of SWE was good for Emean (ICC 0.94, 95% CI 0.86–0.98) and fair for Emax (ICC 0.85, 95% CI 0.66–0.94). Intra- and interobserver agreement between inexperienced and experienced observers showed good to excellent agreement with all ROI methods. Conclusion: Interobserver agreement is high in SWE when performed in a clinical setting. We found the best agreement using the mean value of several ROIs. Intra- and interobserver agreement was high regardless of operator experience.

## 1. Introduction

Endorectal ultrasound (ERUS) is one of the modalities used in the classification of rectal cancer [1]. Ultrasound elastography (UE), providing information on tissue stiffness, can be added to improve the accuracy [2]. An ERUS learning curve exists with a minimum of 50 procedures recommended to achieve a satisfactory level of experience [3,4,5]. Studies suggest low variability between observers based on the investigation of intra- and interobserver agreement of UE [6,7,8]. These studies, however, are retrospective, with two or more observers reassessing previously obtained images. Knowing that ultrasound procedures are observer-dependent and that UE is added during the procedure, it is fair to assume that the variability in UE may increase if investigated in a clinical setting. UE provides information on tissue stiffness by introducing tissue deformation. Currently, two methods of elastography are available in combination with ERUS, i.e., strain elastography (SE) and shear wave elastography (SWE) [7,8,9,10,11,12,13]. While performing SE, the deformation is created either by hand pressure and release or by cardiac or respiratory motion. It is difficult to capture the same information among different acquisitions and users, which may affect the clinical utility. In SWE, a standardized push pulse, produced by the transducer, induces tissue deformation, which creates shear waves that travel through the tissue laterally. In soft tissue, shear waves travel slowly while the speed increases with tissue stiffness. The propagation of shear waves is mapped as a color-coded image on top of a B-mode image to allow the operator to place the region of interest (ROI) optimally [14]. Low variability between observers is important, as tumor classification has a significant impact on treatment planning. In other organs, such as the breast, prostate, liver, and superficial lymph nodes, SWE is reported to have high reproducibility within and across observers [15,16,17,18,19]. However, SWE performed within the rectum may vary if investigated in a clinical setting. To our knowledge, studies investigating interobserver variability in SWE of rectal lesions have only been conducted in an offline setting [7,8]. Performing ERUS has a learning curve, but operator experience in SWE assessment of rectal lesions has not yet been investigated.

Therefore, the purpose of this study was to investigate the agreement on real-time images between two observers that independently perform ERUS SWE assessment of rectal lesions. Furthermore, we aimed to assess the intra- and interobserver agreement of ERUS SWE between experienced and inexperienced observers in an offline setting.

## 2. Materials and Methods

This study was approved by The Regional Committee on Health Research Ethics for Southern Denmark (S-20190176) and registered with ClinicalTrials.gov (NCT04409990). Participation was voluntary, and all patients received oral and written information before signing a consent form.

In order to investigate agreement between two operators in a clinical setting as well as the agreement between experienced and inexperienced observers, we divided the patients into two groups. Patients in Group 1 were investigated by two operators, who, in turn, performed ERUS and SWE. In Group 2, experienced and inexperienced observers reassessed the images of patients who had previously undergone ERUS and SWE examinations. 

### 2.1. Patients

#### 2.1.1. Group 1—Real-Time Interobserver Agreement between Two Experienced Operators

Images were obtained prospectively between June 2021 and December 2021. Only adult patients with a rectal adenoma or adenocarcinoma located <15 cm from the anal verge were included with examinations performed on days when both operators were present in the clinic. During the inclusion period, 43 patients were examined, of which 20 on days with both operators present. One patient was excluded due to pain, leaving a total of 19 patients for analysis.

#### 2.1.2. Group 2—Offline Intra- and Interobserver Agreement between Experienced and Inexperienced Observers

Images obtained from October 2020 to June 2021 were retrospectively assessed. The main inclusion criteria were availability for reassessment on the ultrasound device lesion separable from the surrounding tissue. Images marred by artifacts and those of poor quality or atypical presentation were excluded. An independent radiologist screened all images, and 39 of 61 were eligible for inclusion.

### 2.2. Study Protocol

All patients underwent ERUS at the Department of Radiology, Lillebaelt Hospital, Vejle, Denmark, for evaluation of the tumor as a complex rectal adenoma or a suspected rectal malignancy prior to treatment planning. Patients were placed in the left decubitus position. An initial digital rectal examination was performed prior to the introduction of the ultrasound transducer. We used a Canon Aplio i800 ultrasound machine (Canon Medical Systems, Otawara, Tochigi, Japan) with an endocavity convex probe (PVT-781VTE 3.6–10 MHz). Images were stored on the ultrasound unit for later reassessment using the unit’s software. Tumors were categorized according to the American Joint Committee on Cancer (AJCC) 8th edition TNM classification system [20], i.e., uT1 tumors were confined to mucosa and submucosa. uT2 tumors invaded the muscularis propria without penetrating the rectal wall, uT3 tumors penetrated the rectal wall and perirectal fat, uT4 tumors invaded the nearby pelvic structures. SWE was applied to provide a shared screen with both a color-coded map superimposed on a B-mode image and a B-mode image allowing the examiner to locate the lesion when placing the region of interest (ROI). All SWE measurements were recorded in kilo Pascal (kPa).

#### 2.2.1. Group 1—Real-Time Interobserver Agreement between Two Experienced Operators

Two operators, a resident (MKL) with two years of experience in ERUS and a colorectal radiologist (SRR) with more than 20 years of ERUS experience, scanned the same patient, in turn, blinded to each other’s findings. Both operators had performed more than 50 ERUS examinations. MKL informed the patient and initiated the procedure. First, an assessment of lesion characteristics, including T and N stage. Then, SWE was switched on, providing shared view with both the B-mode image and SWE color map. Three round ROIs were placed within the lesion, the mean (Emean) and maximum (Emax) values were noted, and the images were stored. SRR then entered the examination room and the transducer, still in position, switched hands. SRR performed a similar examination assessing T and N stage and placed three SWE ROIs within the lesion and stored the images before ending the procedure. 

#### 2.2.2. Group 2—Offline Intra- and Interobserver Agreement between Experienced and Inexperienced Observers

**Figure 1:**
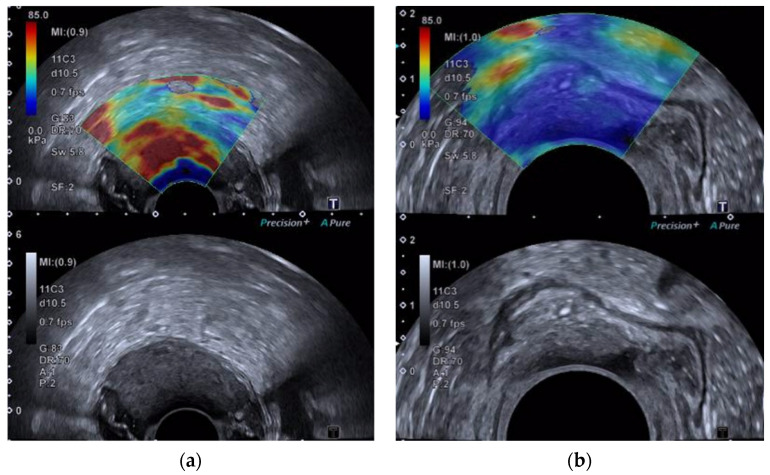
Shear wave elastography images of (**a**) a malignant rectal tumor pT3 and (**b**) a benign rectal polyp. Images are shown on a shared screen with a B-mode image overlaid with a color-coded map where red and yellow represent hard elastography values and blue and green represent soft tissue values. The corresponding B-mode is shown in the lower image.

Four observers assessed the image sequences stored on the ultrasound unit: two residents in radiology (observers 1 and 2) without prior experience in ERUS and two radiologists with >10 years of ERUS experience (observers 3 and 4). An independent radiologist not participating as an observer removed all previous measurements before each read. Images were presented to the observers, as shown in Figure 1. They were instructed to place one round ROI (round ROI) of 1 cm in diameter within the lesion and subsequently place 3 round ROIs with a diameter of 0.3 cm in the most suspicious areas of the lesion. The maximum elastography value (Emax) and the mean value (Emean) were noted in kPa. All observers reassessed the images after three months to reduce recall bias, and they were blinded to each other’s findings, patient information, previous assessments, and radiological and pathological findings. 

### 2.3. Statistics

All statistical analyses were performed using Stata (version 17.0, Stata Corp., College Station, TX, USA). Intra- and interobserver agreement was calculated using two-way fixed absolute agreement models to assess the interclass correlation coefficient (ICC). ICCs were interpreted as poor (<0.5), fair (0.51–0.75), good (0.76–0.90), and excellent (>0.90) [21]. Interobserver agreement of categorical data was calculated using weighted Cohen’s kappa. Kappa values were interpreted as slight 0.00–0.20, fair 0.21–0.40, moderate 0.41–0.60, substantial 0.61–0.80, and almost perfect 0.81–1.00 [22]. Corresponding 95% confidence intervals (CI) were calculated using bootstrapping. For intraobserver agreement, two-way fixed absolute agreement models were used to assess ICC. Bland Altman plots were used to show the difference between two readings against the mean of each test-retest measurement pair. 

## 3. Results

### 3.1. Group 1—Real-Time Interobserver Agreement between Two Experienced Operators

A total of 19 patients were included in Group 1. Pathology reports showed five adenomas and 14 adenocarcinomas. Thirteen patients underwent surgical removal, and the pathology report showed five adenomas, three pT1, one pT2, and four pT3 tumors. Of the remaining six tumors, three were ulT2, and three were ulT3. The median age was 68.5 years ranging from 50–87 years. Males accounted for approximately 60% (11/19).

Interobserver agreement between the two readers was good to excellent for T stage with a weighted kappa of 0.86 (95% CI: 0.71–1.00) and an absolute agreement of 84%, poor to fair for N stage with a kappa of 0.73 (95% CI: 0.35–1.11). Agreement of SWE was good to excellent for the Emean of three ROIs with an ICC of 0.94 (95% CI: 0.86–0.98) and fair to good for the Emax of three ROIs with an ICC of 0.85 (95% CI: 0.66–0.94). Details are shown in Table 1.

### 3.2. Group 2—Offline Intra- and Interobserver Agreement between Experienced and Inexperienced Observers

Thirty-nine patients were eligible for intra- and interobserver variability assessment, of which 21 had adenoma and 18 had adenocarcinoma. Pathology reports showed five pT1, two pT2, five pT3, and one pT4. Of the remaining five adenocarcinomas, four were ulT3 and one ulT4. The median age was 71 years (range 37–98), and males accounted for approximately two-thirds (25/39). 

The interobserver agreement in the first read was good for round ROI as well as the Emean and Emax. In the second read, it was excellent, good, and good, respectively. Intraobserver agreement for observer 1 was excellent regardless of method of ROI; for observers 2 and 3, it was good to excellent, and for observer 4, it was good regardless of ROI method. Detailed ICC values are shown in Table 2.

**Figure 2:**
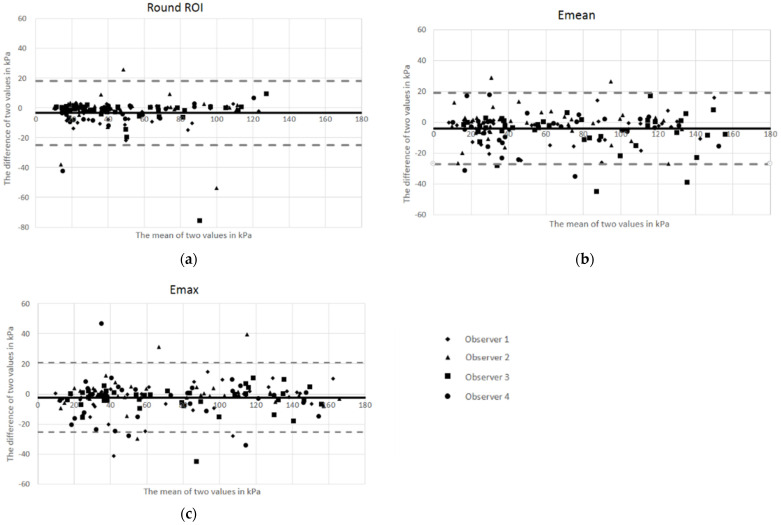
Bland Altman plots showing intraobserver agreement. The *x*-axis shows the average of reads 1 and 2, and the *y*-axis gives the mean of the two reads. The solid horizontal line corresponds to the mean difference (bias). The dotted horizontal lines represent the limits of agreement (LoA): (**a**) Round ROI, elastography value of a round region of interest (ROI) with a diameter of 1 cm; (**b**) Emean, the mean of three small ROIs; (**c**) Emax, the maximum elastography value of three small ROIs.

There was no systematic bias, as shown in the Bland Altman plots (Figure 2). The mean difference in round ROI, Emean, and Emax between the two readings were −3.4 kPa, −3.8 kPa, and −2.4 kPa, respectively. Limits of agreement (LoA) for assessment of round ROI, Emean, and Emax ranged from 18.2 to −25.0 kPa, 19.2 to −26.9 kPa, and 20.8 to −25.5 kPa, respectively. The mean difference in all ROI methods was close to 0, indicating excellent absolute agreement.

## 4. Discussion

This study showed good interobserver agreement when performing SWE measurements in a clinical setting. The most reproducible SWE values were obtained using the mean of three ROIs. Agreement on tumor classification was substantial (kappa 0.86) between the two experienced observers, with an absolute agreement of 84%, which is in accordance with current guidelines [4]. Similar to our findings, Chen et al. [8] found good reproducibility of ERUS T stage assessment with a weighted kappa of 0.76 and excellent agreement of SWE measurements with an ICC of 0.95 between two experienced observers. Li et al. [7] assessed interobserver agreement in ERUS T stage with a kappa value of 0.83. They investigated interobserver agreement of SWE images using a visual scale where predominantly red and yellow colors were considered an indication of malignancy and green and blue a benign lesion. Waage et al. [6] found high reproducibility of intra- and interobserver evaluation of rectal tumors, although they performed strain elastography (SE) using two visual analog scoring systems. In contrast to these studies, our findings were based on real-time images individually obtained by each observer. Our results indicate that the reproducibility of SWE measurements is good between observers in a clinical setting.

Our offline setting showed that obtaining rectal SWE values is independent of observer experience. Observers without ERUS experience performed with high intraobserver agreement as well as experienced observers. This could indicate that SWE may assist upcoming ERUS operators, but the issue needs further investigation. 

Based on Emax as well as Emean, our study showed high agreement between observers in assessed offline images, but in the real-time investigation, we found the highest agreement using the Emean. This difference between the two groups may be explained by the way the images were obtained. In contrast to offline images, real-time images are obtained directly by the observer, and thereby operator dependent in terms of the individual still-image freeze position, the amount of pressure on the lesion, the time until a steady SWE image is presented, and the ROI placement. The mean of several ROIs is therefore preferable to increase clinical observer reproducibility.

### Strengths and Limitations

Our study has several strengths. We included adenomas and adenocarcinomas since patients referred for clinical ERUS evaluation present with either complex polyps or rectal malignancies. The age and sex distribution was similar in the two groups, and in the real-time set-up, two operators scanned the same patient a few minutes apart. While the images were obtained separately, the patient was presented in the same way as to depletion, accessibility, pain, artefacts, etc. Moreover, different ROI methods were applied. We considered it a strength using both experienced and inexperienced observers in our study. To our knowledge, this is the first study to do that.

The low number of patients included is a limitation, as statistical calculations would be strengthened with a larger sample size. In a clinical setting, it can be difficult to have two operators present at the same time during patient examination, especially given the circumstances of the COVID-19 pandemic. 

## 5. Conclusions

We found a high agreement between observers of both T and N stage and SWE values performed in a clinical setting. The highest agreement was found using the mean of three ROIs compared to the maximum value. We found no difference between experienced and inexperienced observers in the evaluation of offline images. 

## Figures and Tables

**Table 1:** Interobserver agreement on tumor T and N stage and shear wave elastography value in Group 1.

Group 1	
Kappa (95% CI) (Agreement)	
T stage *	0.86 (0.71–1.00) (84%)
N stage	0.73 (0.35–1.11) (89%)
ICC (95% CI)	
Emean	0.94 (0.86–0.98)
Emax	0.85 (0.66–0.94)

CI, confidence interval; ICC, interclass correlation coefficient, * weighted kappa; Emean, mean elastography value of three regions of interest; Emax, maximum elastography value of three regions of interest.

**Table 2:** Interclass correlation coefficients of inter- and intraobserver agreement in Group 2.

ICC (95% CI) Region of Interest				
Interobserver		Round ROI (1 cm)	Emean (0.3 cm)	Emax (0.3 cm)
First Read		0.93 (0.89–0.96)	0.92 (0.88–0.96)	0.94 (0.90–0.96)
Second Read		0.95 (0.92–0.97)	0.94 (0.88–0.97)	0.94 (0.90–0.96)
Intraobserver	Experience			
Observer 1	None	0.98 (0.93–0.99)	0.97 (0.94–0.99)	0.97 (0.94–0.98)
Observer 2	None	0.92 (0.86–0.96)	0.96 (0.92–0.98)	0.97 (0.95–0.99)
Observer 3	>10 years	0.92 (0.85–0.96)	0.95 (0.89–0.98)	0.98 (0.95–0.99)
Observer 4	>10 years	0.91 (0.82–0.95)	0.94 (0.88–0-97)	0.94 (0.89–0.97)

ICC, interclass correlation coefficient; CI, confidence interval; Round ROI, elastography value of one round region of interest (ROI) with the diameter of 1 cm; Emean, mean elastography value of three small round ROIs; Emax, maximum elastography value of three small round ROIs.

## Data Availability

The data presented in this study are available on request from the corresponding author.
